# Supplementary material for: Extraction and Purification of Flavonoids from Buddleja officinalis Maxim and Their Attenuation of H2O2-Induced Cell Injury by Modulating Oxidative Stress and Autophagy
Source: Molecules. 2022 Dec 16;27(24):8985. doi: 10.3390/molecules27248985 (PMC9784229; doi:10.3390/molecules27248985)
Supplement: Supplementary file 1 [file molecules-27-08985-s001.zip › molecules-2011582-supplementary.pdf]

Table S1. Other details of luteolin, apigenin and acacetin by HPLC detection.

Number	Phytochemical	Standard curve equation	R^2	linear range (ppb)	LOD (ppb)	LOQ (ppb)
**1**	luteolin	y = 1.2878x − 68.212	0.99992	31.25–1000	3.91	31.25
**2**	apigenin	y = 36.535x − 154.05	0.9998	31.25–1000	3.47	31.25
**3**	acacetin	y = 27.053x + 9.3437	0.99982	3.09–62.5	0.39	3.09
